# A Novel generalization of sequential decision-theoretic rough set model and its application

**DOI:** 10.1016/j.heliyon.2024.e33784

**Published:** 2024-06-28

**Authors:** Tanzeela Shaheen, Hamrah Batool Khan, Wajid Ali, Shaheryar Najam, Md. Zia Uddin, Mohammad Mehedi Hassan

**Affiliations:** a Department of Mathematics, Air University, PAF Complex E-9 Islamabad, 44230, Pakistan; b Department of Electrical Engineering, Riphah International University, Islamabad, Pakistan; c Software and Service Innovation, SINTEF Digital, Oslo, 0373, Norway; d Information Systems Department, College of Computer and Information Sciences, King Saud University, Riyadh, 11543, Saudi Arabia

**Keywords:** Three-way decision, Sequential decision-theoretic rough sets, Optimization, Decision-theoretic rough sets, Decision making

## Abstract

This paper introduces a refined and broadened version of decision-theoretic rough sets (DTRSs) named Generalized Sequential Decision-Theoretic Rough Set (GSeq-DTRS), which integrates the three-way decision (3WD) methodology. Operating through multiple levels, this iterative approach aims to comprehensively explore the boundary region. It introduces the concept of generalized granulation, departing from conventional equivalence-relation-based structures to incorporate similarity/tolerance relations. GSeq-DTRS addresses the limitations encountered by its predecessor, Seq-DTRS, particularly in managing sequential procedures and integrating new attributes. A notable advancement is its seamless handling of continuous-scale datasets through a defined Generalized Granular Structure (GGS), enabling classification across multiple levels without necessitating reduction of attributes. A refined version of conditional probability (CP), aligned with tolerance classes, enhances the approach, supported by a meticulously designed algorithm. Extensive experimental analysis conducted on two datasets sourced from https://www.kaggle.com demonstrates the efficacy of the procedure for both continuous and discrete datasets, effectively classifying probable elements into POS or NEG regions based on their membership. Unlike traditional Seq-DTRS, it does not require reduction of attributes at each new level. Additionally, the algorithm exhibits lower sensitivity to parametric values and yields results in fewer iterations. Thus, its potential impact on decision-making processes is readily apparent.

## 1 Introduction

Uncertainty-related problems are currently receiving more attention from researchers worldwide [[1], [2], [3], [4]]. Consequently, the useful extraction of knowledge from ambiguous data has become a crucial area of study. As a result, numerous approaches, such as rough set theory (RST) [5], have been proposed to analyze ambiguous information. Rough set theory elucidates complex data by discerning essential patterns, facilitating decision-making in uncertain or imprecise environments [6].

Considering the achievements of rough set theory, Yao furthered this framework and introduced the concept of 3WD [7]. Three-way decisions offer a nuanced approach to decision-making, enabling more comprehensive analyses and enhanced adaptability in complex scenarios. Let us examine salient features of these ideas.

RST presented by Pawlak [5] is a valuable model for dealing with data granularity and seeks to address issues based on ambiguity and uncertainty. An ultimate concept of RST is that an indiscernibility relation (R) on the set of objects is based on data analysis. If two objects have identical values for a collection of attributes, they are indiscernible for those attributes. An equivalence relation-based granule is made up of objects with the same description, and a set of all equivalence classes over U forms this universe's partition. These equivalence classes are then employed to generate a pair of sets known as lower and upper approximations of a set X representing a concept, where X⊆U.

### 1.1 Motivation of the study

In 2009, Yao introduced a Three-Way Decision (3WD) theory, which stems from rough set theory [7]. The core concept of 3WD is to break a universe into three regions (X), NEG(X), and BND(X), which are referred to as the “positive”, “negative”, and the “boundary” regions. The positive region comprises elements that decisively belong to a certain category or class, while the negative region consists of elements definitively excluded from that category. The boundary region encompasses elements that are ambiguous or uncertain, requiring further analysis to determine their classification. This idea is often used in human decision-making [[7], [8], [9], [10]]. There has been a spike in interest in the theory and implementation of 3WD in recent years. For example, numerous areas of research might be classified as 3WD space [11,12], three-way (3W) classifications [[13], [14], [15]], 3W concept analysis [12,16,17], 3WD with game theory [17,18], 3W recommender systems [[18], [19], [20]], 3WD support systems [21], fuzzy sets (FSs) 3W approximations of [7], three-valued logic 3W approximations [22], 3W financial decisions [23], Seq-DTRS [8,24,25], dynamic 3WD [25], and many other. These findings provide fresh opportunities for 3WD research and its importance.

Several alternative representations of 3WDs have been proposed to achieve the desired three regions [13,17,26,27]. Among these successful approaches are decision-theoretic rough sets [[28], [29], [30]]. Decision-theoretic rough sets (DTRS) represent a data analysis method that extends the principles of rough set theory and integrates elements of decision theory. In DTRS, a pair of thresholds (α,β) regulate the impurity level within the three regions, facilitating data analysis, object classification, and decision-making in uncertain contexts. It employs a loss function to quantify the cost associated with various classification errors. The efficacy of DTRS heavily depends on the accuracy of the loss function and the data quality utilized for constructing the information system.

A deep study reveals certain limitations of the theories of 3WD and DTRSs, and the literature review shows that it has yet to be studied as a machine learning classification technique [31,32]. Particularly speaking, it cannot be applied to a wide range of case studies for the following reasons: First, the model is designed to classify only those instances that are in the information system. That is to say, this model cannot accommodate an object if its features differ slightly from the labeled (whose decision attribute value is known) objects. This flaw makes it impossible to utilize DTRSs as a classification technique. The second and most significant flaw is that a considerable fraction of objects may fall into the boundary region, and these values remain undecided. This characteristic of the model makes it impractical. For example, in email systems, messages are categorized as either landing in the inbox, not reaching the inbox, or being diverted to the spam folder. In both scenarios, decisions are based on three possibilities: yes, no, or a gray area in between. Ongoing research aims to better understand this gray area. We have discovered that elements within it can be classified based on their likelihood of fitting into one category or another. Our study has explored this idea further. However, as we dive deeper into classification, the risks and potential losses tend to increase.

Yao introduced Seq-DTRS with granular computing to resolve this issue [25] by taking into account the decision process. In Seq-DTRS elements in BND region are to be classified at different iterations while reducing the attributes at those iterations. Seq-DTRS, through granular computing, provides a cost-effective way to make a decision. The decision-making process has crucial factors to consider, as there is a trade-off between the two. Li et al. utilized the concept of Seq-DTRS to create a three-way concept formation axiomatic method [16,24,33,34]. Utilizing a Seq-DTRS strategy and performing sequential tests of statistical hypotheses can result in fast and efficient decision-making methods. Therefore, the implementation of a sequence of 3WD can be applied to medical decision-making, but the process of reducing attributes at each level of GS information is lost, and the results are unreliable.

This study aims to substantially enhance data granulation techniques, ensuring more reliable and widely applicable results. By testing the developed algorithm on real datasets, we seek to showcase its efficiency and effectiveness, facilitating broader adoption and implementation across various domains for decision making [[35], [36], [37]].

### 1.2 Research gap and contributions of this study

Inspired and motivated by the preceding discussion in this article, we find it essential to explore various routes within the multilayered granular framework to fully understand the potential of Seq-DTRS. In the following sections, we highlight the limitations of Seq-DTRS and explain how our study addresses them.

1. Seq-DTRSs use an equivalence relation for granulating elements, requiring exact equivalence among elements under a given relation. However, in real-world granulation scenarios, objects with slight differences are often considered similar and should be grouped together. Equivalence relations do not accommodate this need, but similarity relations do. Therefore, our granulation model employs similarity relations. Additionally, the concept of CP needs to be replaced with a more suitable measure due to the definition of generalized granules. Yao introduced a generalized probability based on tolerance classes [37], which we plan to integrate into our algorithm. We will use this measure in our approach.
2. A major limitation in current studies on Seq-DTRS is the requirement to subtract attributes at each new level. Although this method might be suitable for certain application domains, it can significantly impact the reliability of results. To enhance the algorithm's applicability to various dataset types, we propose a new threshold-based approach where attributes remain consistent at each level. Consequently, each level uses data from all available attributes. This new method is called GSeq-DTRS.
3. The developed algorithm is subsequently tested on real datasets to demonstrate its efficiency and effectiveness.

The following points encapsulate the novelty of the method presented in this paper.

1. GSeq-DTRS offers an enhanced integration of 3WD methodology.
2. The generalized granular structure better accommodates real-world scenarios where objects exhibit slight differences.
3. Introduction of the GGS enables GSeq-DTRS to handle continuous-scale datasets without the need for attribute reduction, addressing a significant limitation of Seq-DTRS.
4. The algorithm presented in this paper exhibits lower sensitivity to parametric values and converges faster, demonstrating improved efficiency and stability.
5. Extensive experimental analysis demonstrates the effectiveness of GSeq-DTRS in classifying elements into POS or NEG regions with fewer iterations compared to traditional Seq-DTRS.

The subsequent parts of the article are structured as follows: Section 2 comprises a brief outline of the concept of 3WDs with DTRS and Seq-DTRS. Section 3 initiates our proposal. This section introduces a fresh approach to sequential DTRSs, along with the presentation of the algorithm. Section 4 is dedicated to the verification and validation of the algorithm via experimental analysis.

## 2 Preliminaries

The current section covers DTRS and GSeq-DTRSs, which are briefly reviewed.

### 2.1 DTRSs

In DTRS, the universe of discourse is divided into rough sets, including positive, negative, and boundary regions, as in traditional rough set theory. However, DTRS utilizes a pair of thresholds on conditional probability to consider decision-making criteria such as utility theory, risk assessment, and decision rules. By incorporating decision-theoretic concepts into rough set analysis, DTRS enable decision-makers to evaluate and compare different decision alternatives in uncertain scenarios [29]. The RST originally approximates a concept by dividing it into the distinct zones: POS, BND, and NEG. The rules generated by the POS region are responsible for acceptance decisions, while the NEG region generates rules for rejection decisions. Finally, the BND zone constructs rules that lead to abstention decisions. These regions partition the universe into three parts that do not overlap and are used to generate rules for classification.

In the context of DTRS, the fundamental principles and concepts of 3WD are presented below.

Consider “an information table S=(U,AT,V,f), where U is a non-empty set of objects, and AT is also a non-empty, finite set such that AT=C∩D, where C and D are disjoint sets representing, respectively, the set of condition attributes and the set of decision attributes. The domain of the attributes is denoted by $V=\underset{a\in AT}{⋃}V_{a}\text{.}$ The function f:U×AT→V is an information function, where $f\left(c_{i},a_{l}\right)$ represents the attribute value of object $c_{i}$ under $a_{l}$ (i=1,2,···,|U|;l=1,2,···,|AT|).”

Let S=(U,AT,V,f) be a 4-tuple and let A⊆C be a subset of attribute. Then, $E_{A}$ is an equivalence relation on U defined [38]:

$$E_{A}=\{\left(c,l\right)\in U\times U\;|\forall a\in A,f\left(c,a\right)=f\left(l,a\right)\}$$

The $E_{A}$ generates a partition of the set U. If c is an object of U, then the set ${\left[c\right]}_{A}=\left\{l\in U\;|\;\left(c,l\right)\in E_{A}\right\}$ represents the equivalence class induced by c and $U/E_{A}=\{\;{\left[c\right]}_{A}|\;c\in U\}$ is the collection of all equivalence classes. Consider X⊆U that shows a particular concept. The CP can be stated as [32]

$$P_{r}\left(X|\left[c\right]\right)=\frac{\left|X\cap \left[c\right]\right|}{\left|\left[c\right]\right|}$$

The symbol |*| is used to represent the cardinality of a set. This CP is equivalent to the rough membership (RM) of c in X. Within the DTRS model, pair of thresholds (α,β) is given with 0<β<α<1, and the (α,β)− probabilistic POS, BND, and NEG regions can be defined as [31]:

$${POS}_{\left(\alpha ,\beta \right)}\left(X\right)=\left\{c\in U|P_{r}\left(X|\left[c\right]\right)\geq \alpha \right\}$$

$${BND}_{\left(\alpha ,\beta \right)}\left(X\right)=\left\{c\in U|\beta <P_{r}\left(X|\left[c\right]\right)<\alpha \right\}$$

$${NEG}_{\left(\alpha ,\beta \right)}\left(X\right)=\left\{c\in U|P_{r}\left(X|\left[c\right]\right)\leq \beta \right\}$$

In order for probabilistic RSs to be utilized in practical applications, it is essential to illuminate the significance of the set of thresholds (α,β), and provide a technique for calculating them. A decision must be made with minimal risk regarding whether to choose the POS, BND, or the NEG region. The DTRS technique consists of two states and three actions. The set of states is denoted by $\Omega =\left\{X,X^{\prime }\right\}\text{,}$ which implies whether an object belongs to X or does not belong to X, respectively, and a set of three actions $Τ=\left\{a_{P},a_{B},a_{N}\right\}\text{,}$ where $a_{P}$, $a_{B}$, and $a_{N}$ show the three actions in classifying an element $-a_{P}$ for c∈POS(X), $a_{B}$ for c∈BND(X), and $a_{N}$ for c∈NEG(X). The loss function, which is concerned to the risk or cost of actions in different states, is represented in a 3×2 matrix in Table 1 [39].

**Table 1:** Decision cost matrix.

	X(P)	$X^{C}\left(N\right)$
$a_{P}\left(P\right)$	$\lambda_{PP}$	$\lambda_{PN}$
$a_{B}\left(B\right)$	$\lambda_{BP}$	$\lambda_{BN}$
$a_{N}\left(N\right)$	$\lambda_{NP}$	$\lambda_{NN}$

Here, the notations $\lambda_{PP}$, $\lambda_{BP}$, and $\lambda_{NP}$ denote the risk or loss incurred for taking the actions $a_{P}$, $a_{B}$ and $a_{N}$, respectively, for elements in X. Likewise, $\lambda_{PN}$, $\lambda_{BN}$ and $\lambda_{NN}$ indicate the risk or loss experienced when doing the similar act for elements in $X^{C}\text{.}$ The expected losses $R\left(a_{i}\;|\left[c\right]\right)$ linked with doing the individual actions can be stated as:

$$R\left(a_{P}|\left[c\right]\right)=\lambda_{PP}P_{r}\left(X|\left[c\right]\right)+\lambda_{PN}P_{r}\left(X^{C}|\left[c\right]\right)\text{,}$$

$$R\left(a_{B}|\left[c\right]\right)=\lambda_{BP}P_{r}\left(X|\left[c\right]\right)+\lambda_{BN}P_{r}\left(X^{C}|\left[c\right]\right)\text{,}$$

$$R\left(a_{N}|\left[c\right]\right)=\lambda_{NP}P_{r}\left(X|\left[c\right]\right)+\lambda_{NN}P_{r}\left(X^{C}|\left[c\right]\right)\text{.}$$

Decision rules with minimum cost can be obtained by using the Bayesian classification procedure [32]:

$$\begin{array}{cc} \left(P\right) & \text{If}\;R\left(a_{P}|\left[c\right]\right)\leq R\left(a_{B}|\left[c\right]\right)\;\text{and}\;R\left(a_{P}|\left[c\right]\right)\leq R\left(a_{N}|\left[c\right]\right),\text{then}\;c\in POS\left(X\right) \end{array}$$

$$\begin{array}{cc} \left(B\right) & \text{If}\;R\left(a_{B}|\left[c\right]\right)\leq R\left(a_{P}|\left[c\right]\right)\;\text{and}\;R\left(a_{B}|\left[c\right]\right)\leq R\left(a_{N}|\left[c\right]\right),\text{then}\;c\in BND\left(X\right) \end{array}$$

$$\begin{array}{cc} \left(N\right) & \text{If}\;R\left(a_{N}|\left[c\right]\right)\leq R\left(a_{P}|\left[c\right]\right)\;\text{and}\;R\left(a_{N}|\left[c\right]\right)\leq R\left(a_{B}|\left[c\right]\right),\text{then}\;c\in NEG\left(X\right) \end{array}$$

Given the constraints $\lambda_{PP}\leq \lambda_{BP}\leq \lambda_{NP},\lambda_{NN}\leq \lambda_{BN}\leq \lambda_{PN}$ and $P_{r}\left(X|\left[c\right]\right)+P_{r}\left(X^{C}|\left[c\right]\right)=1$, the mentioned decision rules are simplified after tie-break as below:

$$\begin{array}{cc} \left(P\right) & \text{If}\;P_{r}\left(X|\left[c\right]\right)\geq \alpha ,\text{then}\;c\in POS\left(X\right)\text{,} \end{array}$$

$$\begin{array}{cc} \left(B\right) & \text{If}\;\beta <P_{r}\left(X|\left[c\right]\right)<\;\alpha ,\text{then}\;c\in BND\left(X\right)\text{,} \end{array}$$

$$\begin{array}{cc} \left(N\right) & \text{If}\;P_{r}\left(X|\left[c\right]\right)\leq \beta ,\text{then}\;c\in NEG\left(X\right)\text{,} \end{array}$$

The DTRS model can be equipped with a loss function that is capable of interpreting and identifying a set of thresholds.

### 2.2 Seq-DTRS

Seq-DTRSs are an extension of DTRSs for sequential DM [32]. The procedure is iterative in nature. It starts with a greater level of granularity and moves down to a lower level. The target is to completely exhaust the boundary region and to classify the elements as belonging to the concept or not. At each new level, attributes are reduced, and less data is used to obtain the results. Below is an explanation of the methodology.

Let $S_{i}=\left(U,{AT}_{i}=C_{i}\cap D,V_{i},f_{i}\right)$ be the information systems, and i=m,...,2,1. Suppose an m− levels granular structure $GS=\left({GS}_{m},.\;.\;.,{GS}_{2},{GS}_{1}\right)\text{,}$ and ${GS}_{i}=(U,E_{i},C_{i},{\;\left[c\right]}_{C_{i}},{\;val\left(c\right)}_{C_{i}}$). At the i−th level of granular structure, it is gained $\left(\alpha_{i},{\;\beta }_{i}\right)-$ probabilistic three regions with DTRS approach in Ref. [33]:

$${POS}_{\left(\alpha ,\beta \right)}^{{GS}_{i}}\left(X_{i}\right)=\left\{c\in U_{i}|P_{r}\left(X_{i}|{\left[c\right]}_{C_{i}}\right)\geq \alpha_{i}\right\}$$

$${BND}_{\left(\alpha ,\beta \right)}^{{GS}_{i}}\left(X_{i}\right)=\left\{c\in U_{i}|\beta_{i}<P_{r}\left(X_{i}|{\left[c\right]}_{C_{i}}\right)<\alpha_{i}\right\}$$

$${NEG}_{\left(\alpha ,\beta \right)}^{{GS}_{i}}\left(X_{i}\right)=\left\{c\in U_{i}|P_{r}\left(X_{i}|{\left[c\right]}_{C_{i}}\right)\leq \beta_{i}\right\}$$

where $X_{i}\in U_{i}$ indicates a concept at the i-th level, $U_{i}$ indicates the elements for managing at the i-th level, $U_{i}\in U$, and $\alpha_{i},\beta_{i}\;\left(0\leq \beta_{i}\leq \alpha_{i}\leq 1\right)$ are the thresholds at the i-th level.

This framework can be well-understood by the following illustration.

Example 1 A prime illustration of three-way decision making is found in medical diagnosis. When a patient presents symptoms to a doctor, the diagnostic process typically entails considering three potential outcomes: the patient may indeed have the suspected condition (POS), the patient may not have the condition at all (NEG), or the symptoms might suggest an alternative underlying issue (BND).

Suppose $U=\left\{c_{1},c_{2},c_{3},c_{4},c_{5},c_{6},c_{7},c_{8},c_{9},c_{10}{,c}_{11},c_{12}\right\}$ is the set of 12 patients that are suspected of COVID, and C={a,b,c} is a set of symptoms (attributes) on U representing fever (a), cough (b), and shortness of breath (SB) (c). Three levels of granularity with the sets of attributes $\left\{C_{1}=\left\{a\right\},C_{2}=\left\{a,b\right\},C_{3}=\left\{a,b,c\right\}\right\}$ are given below.

Level-3: $U/C_{3}=\left\{c_{1},c_{2},c_{3},c_{4},c_{5},c_{6},c_{7},c_{8},c_{9},c_{10}{,c}_{11},c_{12}\right\}$.

Level-2: $U/C_{2}=\{\{c_{1},c_{2},c_{3},c_{4}\},\{c_{5},c_{6},c_{7},c_{8},c_{9},c_{10}\},\{c_{11},c_{12}\}\}$.

Level-1: $U/C_{1}=\{\{c_{1},c_{2},c_{3}\},\{c_{4}\},\{c_{5},c_{6},c_{7}\},\{c_{8},c_{9},c_{10}\},\{c_{11},c_{12}\}\}$.

For the sake of simplicity, $c_{ij}$ will denote the jth equivalence class in the partition $U/C_{i}$. Given a concept $X=\left\{c_{i}|COVID\left(c_{i}\right)=YES\right\}=\left\{c_{1},c_{2},c_{3},c_{5},c_{7}\right\}$ and thresholds $\left(\alpha_{3},\alpha_{2},\alpha_{1}\right)=\left(0.8,0.7,0.6\right)$ and $\left(\beta_{3},\beta_{2},\beta_{1}\right)=\left(0.2,0.3,0.4\right)\text{;}$ the process of Seq-DTRS for level-3 ${GS}_{3}$ is given as below:

Level-3 $X_{3}=X,U_{3}=U$, $U_{3}/C_{3}=c_{31}$. The CP for this level is $P_{r}\left(X_{3}|\left[c_{31}\right]\right)=0.416$.

Then (0.8,0.2)− probabilistic three parts are:

$${POS}_{\left(0.8,0.2\right)}^{{GS}_{3}}\left(X_{3}\right)=\left\{c\in U_{3}|P_{r}\left(X_{3}|{\left[c_{31}\right]}_{C_{3}}\right)\geq 0.8\right\}=\emptyset$$

$${BND}_{\left(0.8,0.2\right)}^{{GS}_{3}}\left(X_{3}\right)=\left\{c\in U_{3}|0.2<P_{r}\left(X_{3}|{\left[c_{31}\right]}_{C_{3}}\right))<0.8\right\}=\left\{c_{1},c_{2},c_{3},c_{4},c_{5},c_{6},c_{7},c_{8},c_{9},c_{10}{,c}_{11},c_{12}\right\}$$

$${NEG}_{\left(0.8,0.2\right)}^{{GS}_{3}}\left(X_{3}\right)=\left\{c\in U_{3}|P_{r}\left(X_{3}|{\left[c_{31}\right]}_{C_{3}}\right)\leq 0.2\right\}=\emptyset$$

Therefore, three accumulated regions are given by

$${POS}^{{GS}_{3}}={POS}_{\left(0.8,0.2\right)}^{{GS}_{3}}\left(X_{3}\right)=\emptyset$$

$${BND}^{{GS}_{3}}={BND}_{\left(0.8,0.2\right)}^{{GS}_{3}}\left(X_{3}\right)=U-{POS}^{{GS}_{3}}-{NEG}^{{GS}_{3}}=\left\{c_{1},c_{2},c_{3},c_{4},c_{5},c_{6},c_{7},c_{8},c_{9},c_{10}{,c}_{11},c_{12}\right\}$$

$${NEG}^{{GS}_{3}}={NEG}_{\left(0.8,0.2\right)}^{{GS}_{3}}\left(X_{3}\right)=\emptyset$$

Similar calculations are made for the accumulated regions at level-2 and level-1. Both are respectively written below.

Level-2 :

$${POS}^{{GS}_{2}}={POS}^{{GS}_{3}}⋃{POS}_{\left(0.7,0.3\right)}^{{GS}_{2}}\left(X_{2}\right)=\left\{c_{1},c_{2},c_{3},c_{4}\right\}$$

$${BND}^{{GS}_{2}}={BND}_{\left(0.7,0.3\right)}^{{GS}_{2}}\left(X_{2}\right)=U-{POS}^{{GS}_{2}}-{NEG}^{{GS}_{2}}=\left\{c_{5},c_{6},c_{7},c_{8},c_{9},c_{10}\right\}$$

$${NEG}^{{GS}_{2}}={NEG}_{\left(0.7,0.3\right)}^{{GS}_{2}}\left(X_{2}\right)=\left\{c_{11},c_{12}\right\}$$

Level-1 :

$${POS}^{{GS}_{1}}={POS}^{{GS}_{2}}⋃{POS}_{\left(0.6,0.4\right)}^{{GS}_{1}}\left(X_{1}\right)=\left\{\left\{c_{1},c_{2},c_{3},c_{4}\right\},\left\{c_{5},c_{6},c_{7}\right\}\right\}$$

$${BND}^{{GS}_{2}}={BND}_{\left(0.7,0.3\right)}^{{GS}_{2}}\left(X_{2}\right)=U-{POS}^{{GS}_{2}}-{NEG}^{{GS}_{2}}=\emptyset$$

$${NEG}^{{GS}_{1}}={NEG}^{{GS}_{2}}⋃{NEG}_{\left(0.7,0.3\right)}^{{GS}_{2}}\left(X_{2}\right)=\left\{c_{11},c_{12}\right\}$$

At this point, a sequence of three regions is obtained using 3 levels of GS, which are as follows:

$${POS}_{sequential}^{GS}=\{level-3:\emptyset ,level-2:\{c_{1},c_{2},c_{3},c_{4}\},level-1:\{c_{5},c_{6},c_{7}\}$$

$${BND}_{sequential}^{GS}=\{level-3:U,level-2:\{c_{5},c_{6},c_{7},c_{8},c_{9},c_{10}\},level-1:\emptyset \}$$

$${NEG}_{sequential}^{GS}=\{level-3:\emptyset ,level-2:\{c_{11},c_{12}\},level-1:\{c_{8},c_{9},c_{10}\}$$

Table 2 illustrates that when considering three attributes in level 3—fever, cough, and shortness of breath—the Seq-DTRS process fails to make definitive decisions for any patient, placing all of them in the boundary region. Consequently, it is unable to identify a single COVID patient. Upon narrowing down to only two symptoms—fever and cough—the procedure labels four patients as COVID-positive and two as negative, which is deemed unreliable due to the limited information. Similarly, the classification of three patients in the positive region and three in the negative region in level-1 lacks reliability as it solely relies on the presence of fever, which could be indicative of various diseases.

**Table 2:** Attributes in each level of seq-DTRS.

	Level-3	Level-2	Level-1
Attributes under each level of GS	{fever, cough, SB}	{fever, cough}	{fever}
Objects in BND	12	6	0
Objects in POS	0	4	3
Objects in NEG	0	2	3

To put it briefly, Seq-DTRS involves reducing attributes at each level of GS, resulting in information loss. In general, if we're examining a disease in an individual, it's crucial to consider as many symptoms or attributes as possible to reach a more accurate conclusion. However, if we continuously decrease the number of symptoms at each level, like in Seq-DTRS, the reliability of our study outcomes diminishes. In below we will present an alternative approach where information remains intact, ensuring no loss of attributes.

## 3 A generalized sequential decision-theoretic rough sets (GSeq-DTRS)

We propose GSeq-DTRS in this article as an extension of the study in Seq-DTRS. The following are the significant novelties of this model: (1) Tolerance/similarity classes have replaced equivalence classes in the hierarchy of granulation. (2) To help with finer granulation of the universe set under consideration, a threshold has been added rather to avoid information loss at each level as in the conventional Seq-DTRS technique. Seq-DTRS will be considerably more widely applicable because reducing attributes at each new level makes the results questionable. (3) A modified version of CP that is perfectly aligned with the tolerance classes has been employed.

We introduce generalized granulation and generalized CP at the beginning of the section. These will serve as the building pieces for the new model.

### 3.1 Generalized granulation structure (GGS)

Based on the information table available, indiscernibility signifies to a situation where it is impossible to differentiate between objects. A reflexive, symmetric, transitive binary relation R defined on a given universe U can be used to represent this situation. Objects of U can be divided into classes, which form the fundamental units of knowledge that can be accessible via R.

The concept of indiscernibility can be extended to include the circumstances in which objects are barely distinguishable from one another. This scenario often arises when the information expressing the elements is inaccurate or, even when it is accurate, when minor variations are irrelevant to the study's objectives. To model this situation using a binary relation, S is defined on U, which signifies a specific type of similarity between the objects.

A fundamental difference with indiscernibility relations is that, generally, similarity relations do not give rise to a partition of the family of elements. Information about similarity can be signified utilizing similarity classes for each object c∈U. More precisely, the similarity class of c, represented by S(c), is a family of objects which are similar to c:

S(c)={y∈U:ySc}

The close association between the basic granules of knowledge is evident in the fact that an object belonging to one similarity class can resemble an object from a different similarity class.

According to Ovchinnikov [40], the concept underlying such a relation is as follows: assume that there are a finite number of objects in Ω and a finite number of their attributes in Δ, and that each object a∈Ω has a minimum of one of each ω∈Δ. Let Ω(ω) be the collection of all a∈Ω with the given attribute ω. Two objects a and b belong to the same subset Ω(ω) for a particular attribute ω∈Δ if and only if we can say that they resemble one another. The formalization of this concept is as follows. Let R be a binary relation on the set Ω defined by aRb if and only if there is ω∈Δ such that a,b∈Ω(ω). R is then a binary reflexive symmetric relation.

Based on the discussion above, a reflexive, symmetric relation can effectively depict how similar two objects are. It can be seen as a method of determining how elements are connected or close to one another. Here, we can build a hierarchical granular structure using a family of similarity classes.

Because they offer a numerical value indicating the level of relationship between two pieces, similarity measures are important. In the current method, we will define similarity class [41] of an object c∈U as follows:

(1) $$S^{\xi }\left(c\right)=\left\{y\in U:S\left(c,y\right)\geq \xi \right\}$$

where,

(2) $$S\left(c,y\right)=1-\sum_{l=1}^{m}\left|f\left(c,a_{l}\right)-f\left(y,a_{l}\right)\right|/\sum_{l=1}^{m}\left|f\left(c,a_{l}\right)+f\left(y,a_{l}\right)\right|$$

ξ is the threshold that determines the level of relationship between c and the objects in $S^{\xi }\left(c\right)$. For all c∈U, the collection of these similarity classes forms a covering of U.

### 3.2 A multilevel granule structure

By placing elements in the POS and NEG regions of a 3WD model, the model determines whether they belong to a concept or not. The boundary region's components are still ambiguous or unclassified. Seq-DTRS was first introduced by Yao [25], as a multi-step process to classify objects in the boundary region. At each stage of this multi-iterative scheme, new information and attributes are required. This requirement restricts the approach's applicability to time-evolving datasets only. We present a GSeq-DTRS based on a generalized granular structure to address this drawback. This framework involves multiple levels of granularity. These similarity relation-based granules are refined using a threshold and there will be no need to decrease attributes and information.

Definition 1 Let IS=(U,AT=C∪D,V,f) be an information system, $S_{i}$ be the similarity relation in (1) and $\xi_{i}$ be the threshold at i−th level, $0<\xi_{i}<1$. Denoting with $S_{i}^{\xi_{i}}$ the similarity class; U/ $S_{i}^{\xi_{i}}$ constitutes a covering of U. The i−th level of generalized granular structure ${GGS}_{i}$ and a multilevel generalized granular structure GGS based on the similarity relation can be represented respectively as:

$${GGS}_{i}=\left(U,S_{i},C,S_{i}^{\xi_{i}},f\right)$$

$$GGS=\left({GSS}_{1},{GSS}_{2},\ldots ,{GSS}_{m}\right)$$

Example 2 Consider an information system in Table 3 with objects $U=\left\{c_{1},c_{2},c_{3},c_{4},c_{5}\right\}$ subject to conditional attributes C={a,b,c}. The last column has been assigned to the decision attribute d.

**Table 3:** Decision matrix.

	a	b	c	d
$c_{1}$	8	45	80	1
$c_{2}$	20	3	100	0
$c_{3}$	40	9	18	1
$c_{4}$	13	17	30	0
$c_{5}$	50	9	15	1

The decision matrix in Table 3 has been normalized in Table 4 with help of the following formula:

$$Ν\left(c_{i}\right)\left(C\right)=\frac{\;f\left(c_{i}\right)\left(C\right)}{\max\;{\;V}_{C}}$$

where $Ν\left(c_{i}\right)\left(C\right)$ is the normalization function, $c_{i}$ are objects from the universal set, $V_{C}$ represents domain values of the attributes C. For example, $Ν\left(c_{3}\right)\left(b\right)=\frac{9}{45}=0.2$.

**Table 4:** Normalized Decision matrix.

	a	b	c
$c_{1}$	0.16	1	0.8
$c_{2}$	0.4	0.0 $\bar{6}$	1
$c_{3}$	0.8	0.2	0.18
$c_{4}$	0.26	0.3 $\bar{7}$	0.3
$c_{5}$	1	0.2	0.15

For the sake of granulation, similarity relation (1) has been employed. Table 5 represents the resulting similarities.

**Table 5:** Similarity matrix.

S	$c_{1}$	$c_{2}$	$c_{3}$	$c_{4}$	$c_{5}$
$c_{1}$	1	0.59922179	0.34394904	0.57822086	0.3081571
$c_{2}$	0.59922179	1	0.48866499	0.52125693	0.43786982
$c_{3}$	0.34394904	0.48866499	1	0.60440714	0.90909091
$c_{4}$	0.57822086	0.52125693	0.604407140	1	0.53326858
$c_{5}$	0.3081571	0.43786982	0.90909091	0.53326858	1

Granulation at **level-1** has been done using Table 5 taking threshold $\xi_{1}=0.59$. The similarity classes are as below.

$$\begin{array}{ccc} S_{1}^{0.59}\left(c_{1}\right)=\left\{c_{1},c_{2}\right\} & S_{1}^{0.59}\left(c_{2}\right)=\left\{c_{1},c_{2}\right\} & S_{1}^{0.59}\left(c_{3}\right)=\left\{c_{3},c_{4},c_{5}\right\} \end{array}$$

$$S_{1}^{0.59}\left(c_{4}\right)=\left\{c_{3},c_{4}\right\}\;S_{1}^{0.59}\left(c_{5}\right)=\left\{c_{3},c_{5}\right\}$$

At **level-2**, granulation with $\xi_{2}=0.6$ would be

$$\begin{array}{ccc} S_{2}^{0.6}\left(c_{1}\right)=\left\{c_{1}\right\} & S_{2}^{0.6}\left(c_{2}\right)=\left\{c_{2}\right\} & S_{2}^{0.6}\left(c_{3}\right)=\left\{c_{3},c_{4},c_{5}\right\} \end{array}$$

$$S_{2}^{0.6}\left(c_{4}\right)=\left\{c_{3},c_{4}\right\}\;S_{1}^{0.6}\left(c_{5}\right)=\left\{c_{3},c_{5}\right\}$$

At **level-3**, taking $\xi_{3}=0.7$, the similarity classes would be

$$\begin{array}{ccc} S_{2}^{0.7}\left(c_{1}\right)=\left\{c_{1}\right\} & S_{2}^{0.7}\left(c_{2}\right)=\left\{c_{2}\right\} & S_{3}^{0.7}\left(c_{3}\right)=\left\{c_{3},c_{5}\right\} \end{array}$$

$$S_{3}^{0.7}\left(c_{4}\right)=\left\{c_{4}\right\}\;S_{1}^{0.7}\left(c_{5}\right)=\left\{c_{3},c_{5}\right\}$$

Here we have done granulation through similarity classes whereas in Example 1, we did granulation through equivalence class. These similarity relation-based granules are refined using a threshold and there will be no need to remove attributes at each iteration.

### 3.3 Generalized conditional probability

For a given set X⊆U, the membership of an element c∈U to a concept X is stated by the following rough membership function (RMF) [42]:

$$\mu_{X}\left(c\right)=\frac{\left|{\left[c\right]}_{R}\cap X\right|}{{\left[c\right]}_{R}}$$

These RMFs can be considered as conditional probabilities that any object belongs to X given that the object belongs to ${\left[c\right]}_{R}$. Here, ${\left[c\right]}_{R}$ represents an equivalence class of c subject to the relation R. Yao et al. [38] designed a parallel measure of membership when a covering of U is given instead of partition. Similarity classes within a covering may overlap one another. The extended versions of RMF for finding membership μ of c to X from the perspective of a similarity class $S_{i}$ of U are given as [37]:

$$\mu_{X}^{m}\left(c\right)=\min\left\{\frac{\left|S_{i}\cap X\right|}{S_{i}}\;|c\in S_{i}\right\}$$

$$\mu_{X}^{M}\left(c\right)=\max\left\{\frac{\left|S_{i}\cap X\right|}{S_{i}}\;|c\in S_{i}\right\}\text{,}$$

$$\mu_{X}^{*}\left(c\right)=\text{avg}\left\{\frac{\left|S_{i}\cap X\right|}{S_{i}}\;|c\in S_{i}\right\}\text{.}$$

These are respectively the minimum, maximum and the average RMF representing the most indulgent, the most optimistic and the most stable measure respectively. These three measures are respectively applied when X has the minimum overlap with $S_{i}$, the maximum overlap and the non-empty intersection with majority of the $S_{i}$ in a given covering of U. The average RMF is most suitable in our case. So, we'll incorporate this in our model. Thus, re-writing it as CP and utilizing the similarity classes defined in (1) we get

(3) $$\mathrm{Pr}\left(X|c\right)=avg\left\{\frac{\left|S_{i}^{\xi_{i}}\cap X\right|}{S_{i}^{\xi_{i}}}\;|c\in S_{i}^{\xi_{i}}\right\}$$

### 3.4 GSeq-DTRS

Having discussed all the required components of our model, we are now ready to devise and discuss it which we refer to as GSeq-DTRS. This section will serve this purpose.

**Fig. 1:**
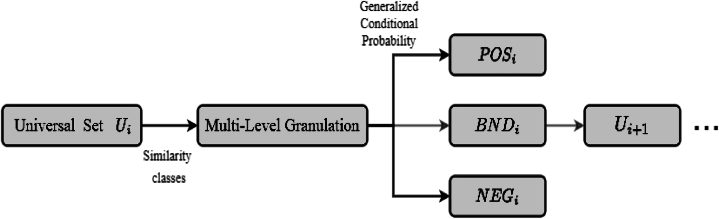
The general model of GSeq-DTRS.

Fig. 1 shows how this model works. Using similarity relation (1), the universe set is divided into similarity classes. The universe set's elements are divided into the POS, NEG, and BND regions at each level of GGS. The process is then repeated using the BND region as the universe set at the following level of GGS. At the i−th level of granular structure, the $\left(\alpha_{i},\beta_{i}\right)-$ probabilistic three regions with GSeq-DTRS model as defined as follows:

$${POS}_{\left(\alpha_{i},\beta_{i}\right)}^{{GGS}_{i}}\left(X_{i}\right)=\left\{c\in U_{i}|\mathrm{Pr}\left(X_{i}|S_{i}^{\xi_{i}}\left(c\right)\right)\geq \alpha_{i}\right\}$$

$${BND}_{\left(\alpha_{i},\beta_{i}\right)}^{{GGS}_{i}}\left(X_{i}\right)=\left\{c\in U_{i}|\beta_{i}<\mathrm{Pr}\left(X_{i}|S_{i}^{\xi_{i}}\left(c\right)\right)<\alpha_{i}\right\}$$

$${NEG}_{\left(\alpha_{i},\beta_{i}\right)}^{{GGS}_{i}}\left(X_{i}\right)=\left\{c\in U_{i}|\mathrm{Pr}\left(X_{i}|S_{i}^{\xi_{i}}\left(c\right)\right)\leq \beta_{i}\right\}$$

$X_{i}$ indicates a concept at the i−th level, ${X\;}_{i}\in U_{i}$ , $U_{i}$ indicates the elements for managing at the i−th level, $\alpha_{i},\beta_{i}$ are pair of thresholds at the i−th level, $0\leq \beta_{i}<\alpha_{i}\leq 1$.

In the process, from the i-th level of GGS to the (i+1)-th level we have ${\;U}_{i+1}={BND}_{\left(\alpha_{i},\beta_{i}\right)}^{{GGS}_{i}}\left(X_{i}\right)$ and $X_{i+1}=X_{i}\cap {BND}_{\left(\alpha_{i},\beta_{i}\right)}^{{GGS}_{i}}\left(X_{i}\right)\text{.}$ Temporarily, we gain a series of POS, BND, and NEG region under m-levels GGS as below:

$${POS}_{sequential}^{GGS}=\left({POS}_{\left(\alpha_{1},\beta_{1}\right)}^{{GGS}_{1}}\left(X_{1}\right),{POS}_{\left(\alpha_{2},\beta_{2}\right)}^{{GGS}_{2}}\left(X_{2}\right),\ldots ,{POS}_{\left(\alpha_{m},\beta_{m}\right)}^{{GGS}_{m}}\left(X_{m}\right)\right)\text{,}$$

$${BND}_{sequential}^{GGS}=\left({BND}_{\left(\alpha_{1},\beta_{1}\right)}^{{GGS}_{1}}\left(X_{1}\right),{BND}_{\left(\alpha_{2},\beta_{2}\right)}^{{GGS}_{2}}\left(X_{2}\right),\ldots ,{BND}_{\left(\alpha_{m},\beta_{m}\right)}^{{GGS}_{m}}\left(X_{m}\right)\right)\text{,}$$

$${NEG}_{sequential}^{GGS}=\left({NEG}_{\left(\alpha_{1},\beta_{1}\right)}^{{GGS}_{1}}\left(X_{1}\right),{NEG}_{\left(\alpha_{2},\beta_{2}\right)}^{{GGS}_{2}}\left(X_{2}\right),\ldots ,{NEG}_{\left(\alpha_{m},\beta_{m}\right)}^{{GGS}_{m}}\left(X_{m}\right)\right)$$

#### 3.4.1 Algorithm

**Figure:**
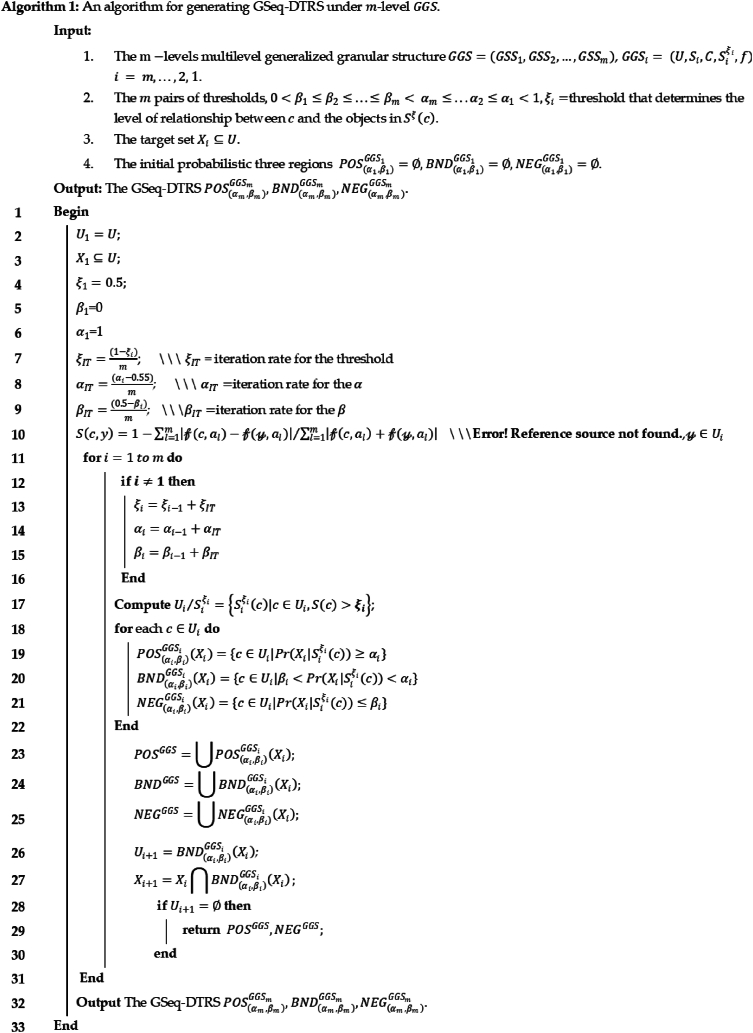

Example 3 Continuing Example 2, at **level 1**, $U_{1}=\left\{c_{1},c_{2},c_{3},c_{4},c_{5}\right\}$ and target set is $X_{1}=\left\{c_{1},c_{3},c_{5}\right\}$. Using formula (2) conditional probabilities are calculated as below.

$$\mathrm{Pr}\left(X_{1}|c_{1}\right)=\frac{\frac{1}{2}+\frac{1}{2}}{2}=0.5,\mathrm{Pr}\left(X_{1}|c_{2}\right)=0.5,\mathrm{Pr}\left(X_{1}|c_{3}\right)=0.7\bar{2},\mathrm{Pr}\left(X_{1}|c_{4}\right)=0.58\bar{3}\;\text{and}\;\mathrm{Pr}\left(X_{1}|c_{5}\right)=0.8\bar{3}$$

With $\beta_{1}=0.2$ and $\alpha_{1}=0.8$ the elements are classified as below.

$${POS}_{\left(\alpha_{1},\beta_{1}\right)}^{G{GS}_{1}}\left(X_{1}\right)=\left\{c_{5}\right\}$$

$${BND}_{\left(\alpha_{1},\beta_{1}\right)}^{{GGS}_{1}}\left(X_{1}\right)=\left\{c_{1},c_{2},c_{3},c_{4}\right\}$$

$${NEG}_{\left(\alpha_{1},\beta_{1}\right)}^{G{GS}_{1}}\left(X_{1}\right)=\emptyset$$

At **level-2**, ${\;U}_{2}={BND}_{\left(\alpha_{1},\beta_{1}\right)}^{{GGS}_{1}}\left(X_{1}\right)=\left\{c_{1},c_{2},c_{3},c_{4}\right\}$, ${\;X}_{2}=X_{1}\cap {BND}_{\left(\alpha_{1},\beta_{1}\right)}^{G{GS}_{1}}\left(X_{1}\right)=\left\{c_{1},c_{2}\right\}$.

Calculating the probabilities and setting $\beta_{2}=0.3$ and $\alpha_{2}=0.7$ we get

$${POS}_{\left(\alpha_{2},\beta_{2}\right)}^{{GGS}_{2}}\left(X_{2}\right)=\left\{c_{1}\right\}$$

$${BND}_{\left(\alpha_{2},\beta_{2}\right)}^{{GGS}_{2}}\left(X_{2}\right)=\left\{c_{3},c_{4}\right\}$$

$${NEG}_{\left(\alpha_{2},\beta_{2}\right)}^{{GGS}_{2}}\left(X_{2}\right)=\left\{c_{2}\right\}$$

At **level-3**, $U_{3}={BND}_{\left(\alpha_{2},\beta_{2}\right)}^{G{GS}_{2}}\left(X_{2}\right)=\left\{c_{3},c_{4}\right\}$, $X_{3}=X_{2}\cap {BND}_{\left(\alpha_{2},\beta_{2}\right)}^{G{GS}_{2}}\left(X_{2}\right)=\left\{c_{3}\right\}$.

With $\beta_{3}=0.4$ and $\alpha_{3}=0.5$ the three regions are

$${POS}_{\left(\alpha_{3},\beta_{3}\right)}^{{GGS}_{3}}\left(X_{3}\right)=\left\{c_{3}\right\}$$

$${BND}_{\left(\alpha_{3},\beta_{3}\right)}^{{GGS}_{3}}\left(X_{3}\right)=\emptyset$$

$${NEG}_{\left(\alpha_{3},\beta_{3}\right)}^{{GGS}_{3}}\left(X_{2}\right)=\left\{c_{4}\right\}$$

Table 6 shows that without loss of information, the boundary region has been completely exhausted in only three iterations. The generalized granular structure as well as the generalized conditional probabilities make this algorithm more flexible. Furthermore, the classification of objects includes their respective probabilities, indicating the certainty of their classification.

**Table 6:** Summary of results via algorithm 1.

	Level-1	Level-2	Level-3
Attributes under each level of GS	{a,b,c}	{a,b,c}	{a,b,c}
Objects in BND	4	2	0
Objects in POS	1	1	1
Objects in NEG	0	1	1

## 4 Experimental analyses

The developed algorithm is applied in python programming language and Nvidia TX2 GPU Development Kit was used with Ubuntu operating system. To acquire maximum performance, the system configuration was set to maximum using the performance governor provided by Ubuntu operating system.

### 4.1 Classification results of stroke prediction dataset

**Fig. 2:**
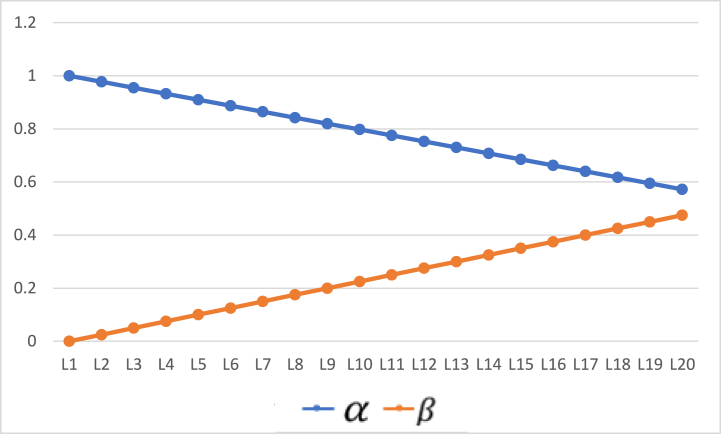
When α = 1, β = 0 (initial).

Results were compiled against various datasets and for deeper analysis the dataset was segregated into two sizes such as 57 and 100 objects. Initially, stroke prediction dataset was used (https://www.kaggle.com/datasets/fedesoriano/stroke-prediction-dataset?select=healthcare-dataset-stroke-data.csv) and the dataset included 11 clinical features for predicting stroke events. Algorithm 1 is applied to the two sets of objects. In this context, the POS region identifies individuals who are confidently predicted to have a stroke, while the NEG region comprises those confidently predicted not to have a stroke. The BND region includes individuals where the evidence is insufficient to confidently categorize them into POS or NEG. The algorithm's objective is to accurately classify individuals in the boundary region with a high probability. Essentially, this entails assigning probable subjects to the region where they are most likely to belong based on their characteristics. The thresholds α,β have been taken with fixed and small step-size 0.0225. For the first set of experiments, we take α=1,β=0. The small change in the threshold values at each level will be helpful in analyzing the algorithm deeply. Fig. 2 gives these values.

The output of algorithm 1 on 57 objects classified in the POS, BND, and NEG regions after 20 iterations is shown in Table 7. The target is to continue iterations till the boundary region becomes empty. That is to say, all the probable elements are classified into positive and negative regions with some probability. Thus, the elements in the boundary region generate decisions at each level under different thresholds. It should be noted that at each level of iteration, a full set of information (attributes) has been utilized and no information loss is required at every next level.

**Table 7:** Objects = 57 α=1β=0 (initial).

	$L_{1}$	$L_{2}$	$L_{3}$	$L_{4}$	$L_{5}$	$L_{6}$	$L_{7}$	$L_{8}$	$L_{9}$	$L_{10}$	$L_{11}$	$L_{12}$	$L_{13}$	$L_{14}$	$L_{15}$	$L_{16}$	$L_{17}$	$L_{19}$	$L_{20}$	$L_{21}$
POS	0	0	0	0	0	0	0	0	0	0	0	0	2	3	0	10	1	3	0	2
BND	57	57	57	57	56	55	55	55	55	53	52	52	48	38	30	13	8	4	2	0
NEG	0	0	0	0	1	1	0	0	0	2	1	0	2	7	8	7	4	1	2	0

It can be seen from Table 7 that the generalized sequential process keeps on decreasing the objects in boundary region while adding them in the positive as well as negative region. The advantage of applying this algorithm is that it guarantees that the boundary region will be exhausted, and no object will remain undecisive.

Further, the proposed algorithm was also evaluated against same data with 100 objects and the results are portrayed in Table 8.

**Table 8:** Objects = 100 α=1β=0 (initial).

	$L_{1}$	$L_{2}$	$L_{3}$	$L_{4}$	$L_{5}$	$L_{6}$	$L_{7}$	$L_{8}$	$L_{9}$	$L_{10}$	$L_{11}$	$L_{12}$	$L_{13}$	$L_{14}$	$L_{15}$	$L_{16}$	$L_{17}$	$L_{18}$	$L_{19}$	$L_{20}$
POS	0	0	0	0	0	0	0	0	0	0	0	0	4	0	3	7	3	0	2	0
BND	57	57	57	57	56	55	55	53	52	50	50	50	43	31	24	11	4	4	0	0
NEG	0	0	0	0	1	1	0	2	1	2	0	0	3	12	4	6	4	0	2	0

**Fig. 3:**
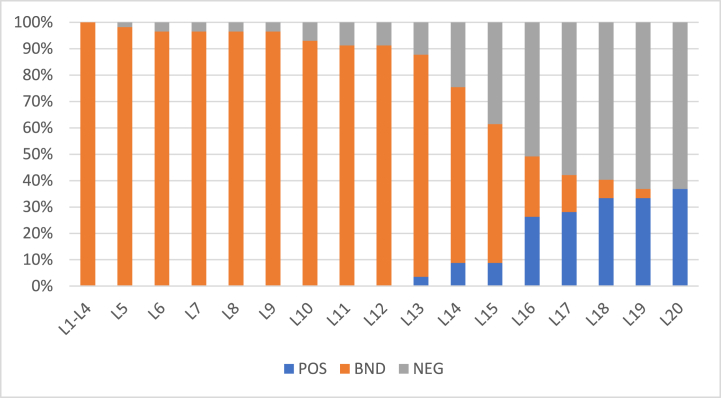
Rate of inclusion of 57 objects in three regions with (α,β)=(1,0) in L1.

**Fig. 4:**
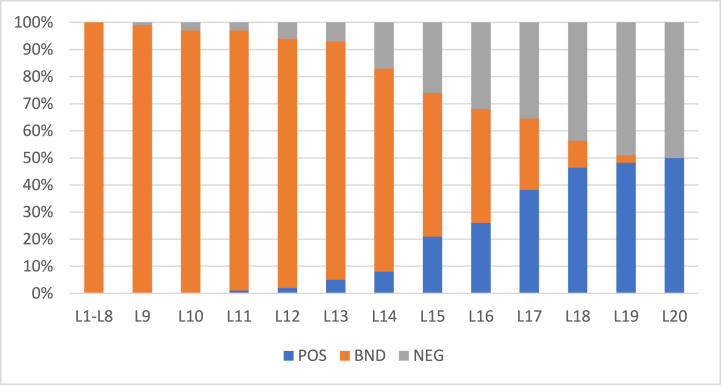
Rate of inclusion of 100 objects in three regions (α,β)=(1,0) in L1.

Fig. 3, Fig. 4 show the rates of object inclusion in three regions for 57 and 100 objects, respectively. In these figures, x− axis represents levels (iterations) and y− axis represents the rate of objects’ inclusion the three regions. With an increase in the values the α the rate of inclusion in boundary region decreases whereas in positive and negative regions it increases. Thus, the rate is monotonic. In level 20, there is no object left in the boundary region.

Additionally, it is evident from the results that increase or decrease in the number of objects do not affect the efficiency of the algorithm. Locally, there are slight differences in the decision rates of objects, yet both the experiments exhaust boundary region in 20 levels.

**Fig. 5:**
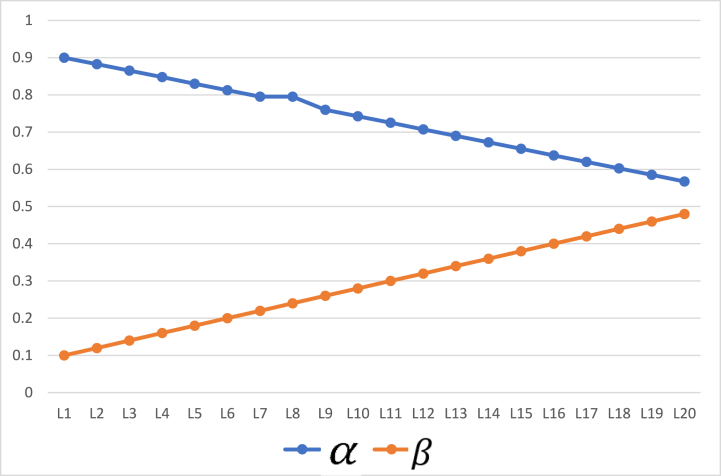
Values of α and β with initial values α = 0.9, β = 0.1.

**Fig. 6:**
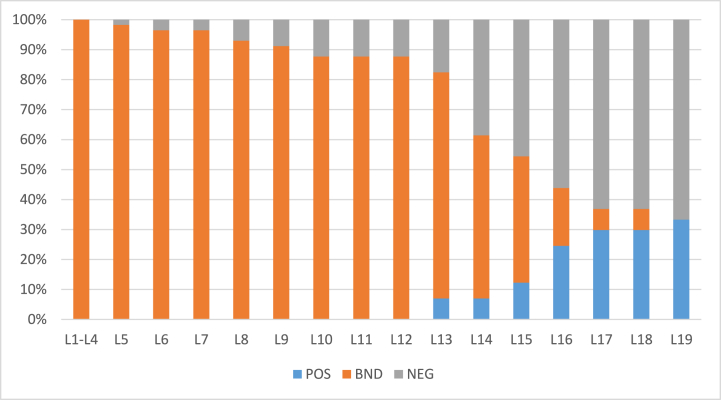
Rate of inclusion of 57 objects in three regions.

**Fig. 7:**
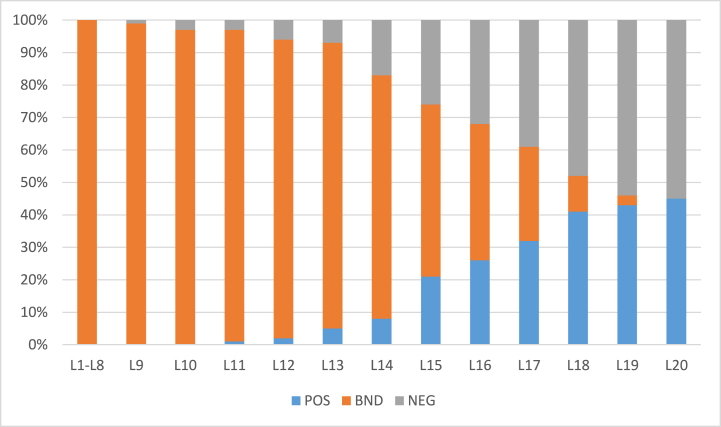
Rate of inclusion of 100 objects in three regions.

To see the effect of change of thresholds on the results, we repeat the experiments on the same sets of objects with step size 0.0175. Furthermore, the initial values of α and β have also been changed and set to 0.9 and 0.1 respectively. Fig. 5 shows these values. Results for both 57 objects and 100 objects are provided in Table 9 and Table 10, respectively. By changing the values of thresholds, the probabilities of objects’ inclusion in positive and negative regions changes. Yet, the rates are monotonic and generate results within 20 iterations. The rates of object inclusion in three regions from 57 to 100 objects are also provided in Fig. 6, Fig. 7, respectively

**Table 9:** Objects = 57 α=0.9β=0.1 (initial).

	$L_{1}$	$L_{2}$	$L_{3}$	$L_{4}$	$L_{5}$	$L_{6}$	$L_{7}$	$L_{8}$	$L_{9}$	$L_{10}$	$L_{11}$	$L_{12}$	$L_{13}$	$L_{14}$	$L_{15}$	$L_{16}$	$L_{17}$	$L_{18}$	$L_{19}$	$L_{20}$
POS	0	0	0	0	0	0	0	0	0	0	0	0	4	0	3	7	3	0	2	0
BND	57	57	57	57	56	55	55	53	52	50	50	50	43	31	24	11	4	4	0	0
NEG	0	0	0	0	1	1	0	2	1	2	0	0	3	12	4	6	4	0	2	0

**Table 10:** Objects = 100 α=0.9β=0.1 (initial).

	$L_{1}$	$L_{2}$	$L_{3}$	$L_{4}$	$L_{5}$	$L_{6}$	$L_{7}$	$L_{8}$	$L_{9}$	$L_{10}$	$L_{11}$	$L_{12}$	$L_{13}$	$L_{14}$	$L_{15}$	$L_{16}$	$L_{17}$	$L_{18}$	$L_{19}$	$L_{20}$
POS	0	0	0	0	0	0	0	0	0	0	3	1	2	2	18	3	5	7	3	3
BND	100	100	100	100	100	100	100	98	98	97	94	90	86	72	48	39	26	15	7	0
NEG	0	0	0	0	0	0	0	2	0	1	0	3	2	12	6	6	8	4	5	4

All the elements in the boundary region are classified in positive or negative regions at the last level.

#### 4.1.1 Classification accuracy

**Fig. 8:**
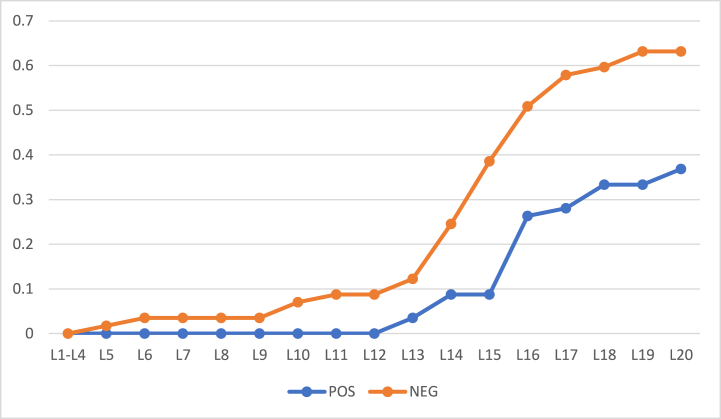
Classification Accuracy of POS and NEG regions for 57 objects with initial thresholds α = 1 and β = 0.

**Fig. 9:**
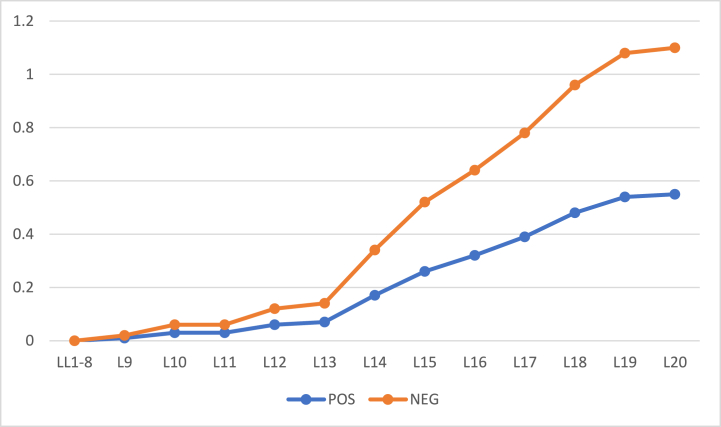
Classification Accuracy of POS and NEG regions for 100 objects with initial thresholds α = 1 and β = 0.

To analyze the accuracy of our algorithm, classification accuracy has been calculated and the results have been displayed in Fig. 8, Fig. 9. It can be seen that the accuracy keeps on increasing at each new iteration.

**Fig. 10:**
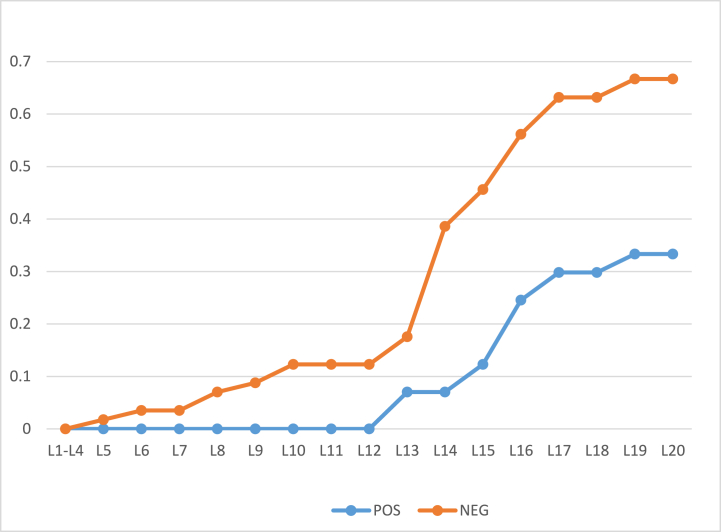
Classification Accuracy of BND region, with 57 objects having α = 0.9 and β = 0.1(initial).

**Fig. 11:**
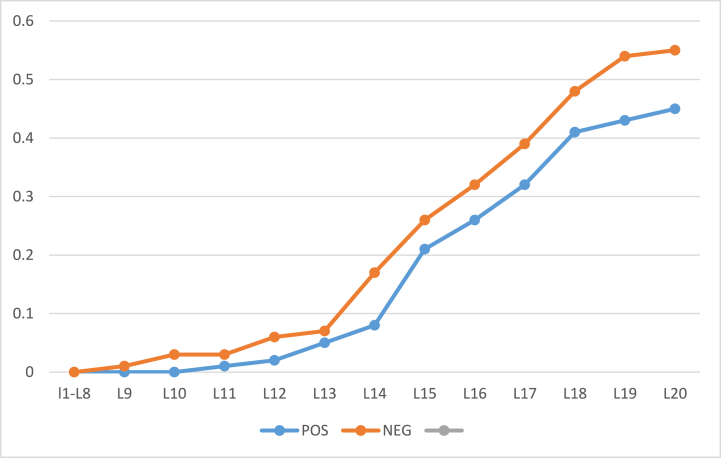
Classification Accuracy of BND region, with 100 objects having α = 0.9 and β = 0.1(initial).

Classification Accuracy of POS and NEG regions with 57 and 100 objects having α=0.9 and β=0.1 (initial) is shown in Fig. 10, Fig. 11.

### 4.2 Classification results of breast cancer dataset

**Fig. 12:**
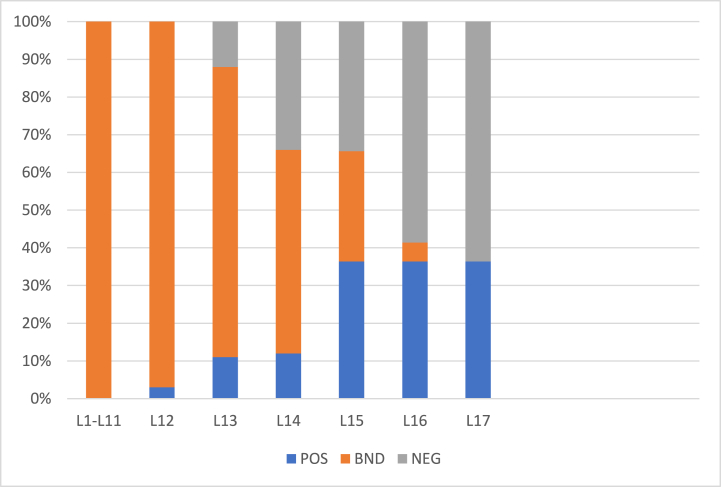
Rate of inclusion of 100 objects in three regions, α=1β=0 (initial).

**Fig. 13:**
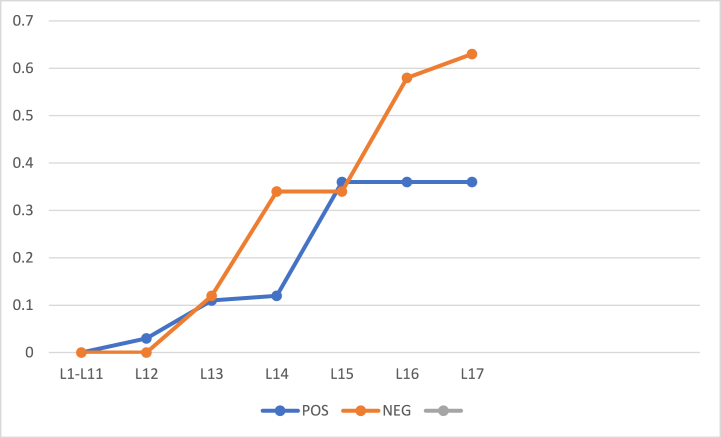
Classification Accuracy of POS and NEG regions, with 100 objects having α=1 and β=0 (initial).

**Fig. 14:**
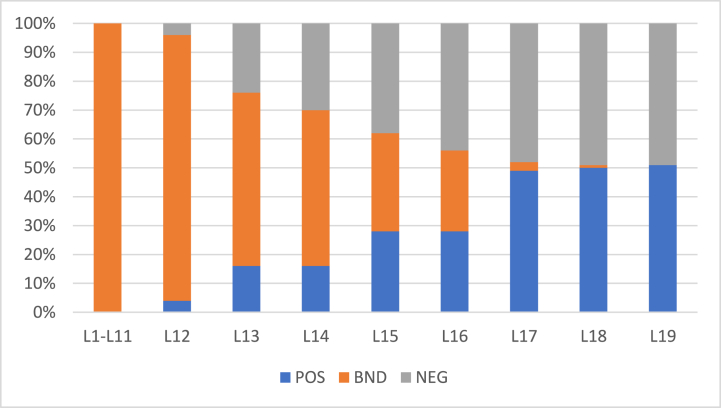
Rate of inclusion of 100 objects in three regions, α=0.9β=0.1 (initial).

**Fig. 15:**
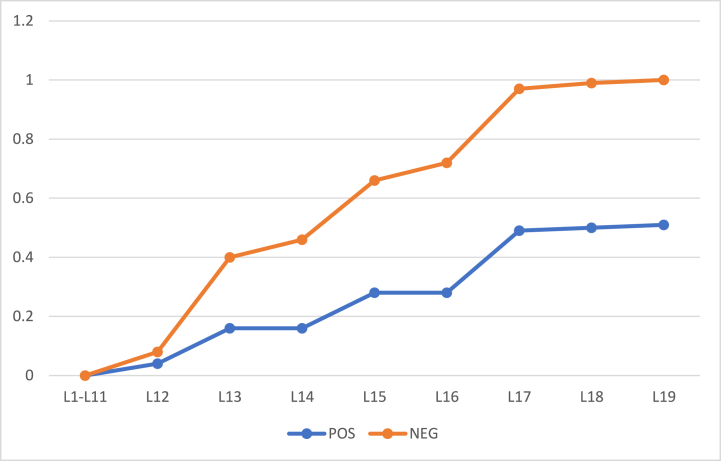
Classification Accuracy of BND region, with 100 objects having α=0.9 and β=0.1 (initial).

The algorithm was also evaluated against another dataset for comparative analysis. The dataset was Breast Cancer Wisconsin (Diagnostic) Data Set to predict whether the cancer is benign or malignant cancer (https://www.kaggle.com/datasets/uciml/breast-cancer-wisconsin-data). The POS region encompasses individuals with benign cancer, while the NEG region comprises those with malignant cancer. The BND region includes subjects where the distinction is less clear, representing probable cases. The algorithm's goal is to accurately classify these subjects based on their proximity to either the POS or NEG region. The results were evaluated for two different values of α and β and shown in Table 11 and Table 12. Further, the rate of inclusion of 100 object with α=1 and β=0 (initial) is presented in Fig. 12 and with α=0.9 and β=0.1 (initial) is presented by Fig. 14 whereas the classification accuracy of their respective POS and NEG regions are presented in Fig. 13, Fig. 15. The results have similar interpretations as in the case of stroke prediction dataset.

**Table 11:** Objects = 100 α=1β=0 (initial).

	$L_{1}$	$L_{2}$	$L_{3}$	$L_{4}$	$L_{5}$	$L_{6}$	$L_{7}$	$L_{8}$	$L_{9}$	$L_{10}$	$L_{11}$	$L_{12}$	$L_{13}$	$L_{14}$	$L_{15}$	$L_{16}$	$L_{17}$
POS	0	0	0	0	0	0	0	0	0	0	0	3	8	1	25	0	0
BND	100	100	100	100	100	100	100	100	100	100	100	97	77	54	29	5	0
NEG	0	0	0	0	0	0	0	0	0	0	0	0	12	22	0	24	5

**Table 12:** Objects = 100 α=0.9β=0.1 (initial).

	$L_{1}$	$L_{2}$	$L_{3}$	$L_{4}$	$L_{5}$	$L_{6}$	$L_{7}$	$L_{8}$	$L_{9}$	$L_{10}$	$L_{11}$	$L_{12}$	$L_{13}$	$L_{14}$	$L_{15}$	$L_{16}$	$L_{17}$	$L_{18}$	$L_{19}$
POS	0	0	0	0	0	0	0	0	0	0	0	4	12	0	12	0	21	1	1
BND	100	100	100	100	100	100	100	100	100	100	100	92	60	54	34	28	3	1	0
NEG	0	0	0	0	0	0	0	0	0	0	0	4	20	6	8	6	4	1	0

In the experimental phase, we immerse ourselves deeply in the intricate process of implementing and meticulously evaluating Algorithm 1 across datasets containing 57 and 100 objects. Our primary aim was to systematically categorize these objects into distinct positive, negative, and boundary regions until the elusive boundary region is completely eliminated. This exhaustive undertaking relies heavily on the precise utilization of a comprehensive set of attributes, eliminating the need for deletion of essential features. The empirical evidence meticulously documented in Table 7, Table 8, Table 9, Table 10 and Fig. 2, Fig. 3, Fig. 4, Fig. 5, Fig. 6, Fig. 7, Fig. 8, Fig. 9, Fig. 10, Fig. 11 emphatically underscores the algorithm's steadfast capability to deplete the boundary realm within a mere 20 iterations, irrespective of the dataset's object density. Moreover, the exploration of various threshold configurations unveils a conspicuously monotonic trend in object inclusion rates across different regions, lending an aura of undeniable conclusiveness to the outcomes. Furthermore, to bolster the algorithm's credibility and universality, it undergoes a rigorous examination when applied to the Breast Cancer Wisconsin (Diagnostic) Data Set. The ensuing analysis, meticulously presented in Table 11, Table 12 and Fig. 12, Fig. 13, Fig. 14, Fig. 15, intricately juxtaposes the algorithm's performance under diverse α and β values against the backdrop of a novel dataset. These nuanced observations attest to the algorithm's immutable efficacy across diverse data landscapes, solidifying its stature as a cornerstone in the domain of computational methodologies.

### 4.3 Comparative analysis

In the realm of Decision-Theoretic Rough Set (DTRS) algorithms, various sequential approaches have been proposed to efficiently handle data classification tasks. In Ref. [33], a sequential approach is introduced, characterized by fixed levels (iterations) that expedite processing but may compromise accuracy due to limited threshold considerations and reduced data utilization. However, this algorithm's reliance on a predetermined number of iterations and fixed thresholds restricts its adaptability and may lead to less reliable results.

In contrast, our proposed algorithm aims to address these limitations by providing flexibility in threshold determination while utilizing all available data to enhance classification accuracy. Unlike the approach in Ref. [33], which is constrained by a fixed number of thresholds based on predetermined alpha and beta values, our algorithm empowers users to define thresholds as needed, enabling a more comprehensive evaluation of data elements and improving the reliability of their classification into positive (POS) or negative (NEG) regions.

Furthermore, the analysis of the algorithm presented in Ref. [33] reveals that the distribution of objects into their respective regions significantly impacts the results. Notably, in Ref. [33], Fig. 5 shows that the distribution of objects to their respective regions highly affect the results. For almost all the datasets, the first iteration that utilizes all the data hardly classifies any object. Thus, with a full set of information, we cannot get our required result from this algorithm. For data set "car", boundary region is not exhausted and after the final iteration, there are still some elements left in the boundary region that remain undecisive. Conversely, our approach ensures that the full set of information is leveraged from the outset, enhancing the effectiveness and reliability of the classification process.

Additionally, the comparison with [43], which employs intuitionistic fuzzy numbers in a sequential DTRS framework, highlights a fundamental departure from traditional DTRS methodologies. By replacing conditional probability with intuitionistic grade [43], introduces a novel perspective that focuses on the membership of elements to concepts rather than their similarity to clusters. While this deviation offers intriguing insights, it underscores the evolving nature of DTRS methodologies and the potential for diverse approaches to address classification challenges.

In conclusion, our proposed algorithm represents a significant advancement in sequential DTRS methodologies by offering enhanced flexibility, improved data utilization, and increased reliability in classification outcomes. By allowing users to define thresholds dynamically and leveraging all available information, our approach provides a more comprehensive and accurate analysis of data elements, thereby expanding the applicability and effectiveness of DTRS in real-world scenarios.

## 5 Sensitivity analysis

The sensitivity of our algorithm, developed through the generalization of sequential decision theoretic rough sets, lies in its utilization of two key parameters, α and β. Initially, the algorithm employs three-way decision theory to classify objects. Subsequently, in successive iterations, it further refines classification within the boundary region by allocating elements to positive and negative regions based on their membership to each.

**Fig. 16:**
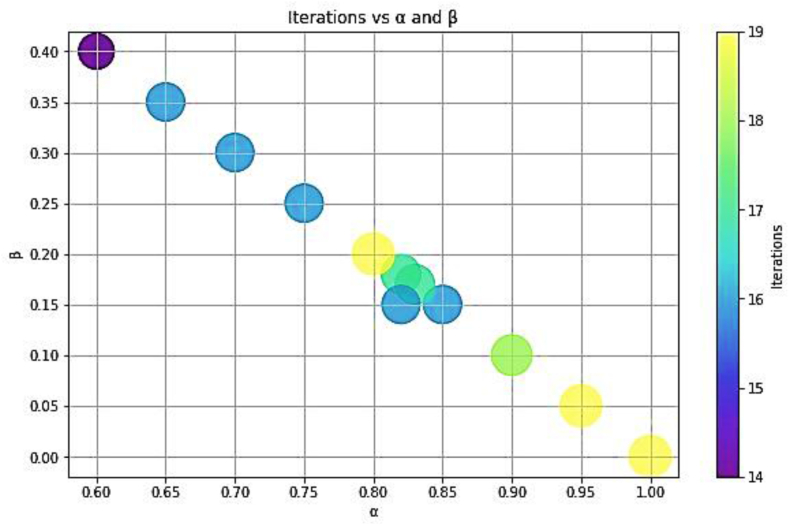
Iterations Vs parameters for heart stroke dataset.

**Fig. 17:**
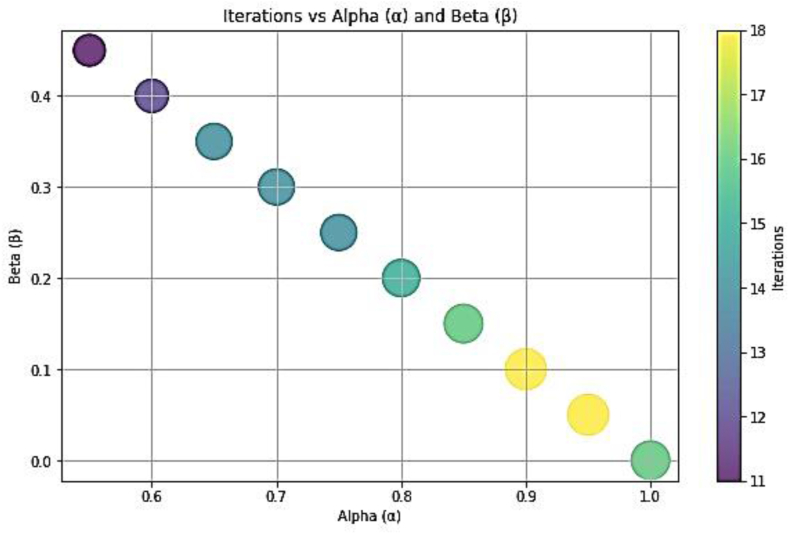
Iterations Vs parameters for breast cancer dataset.

The determination of membership relies on conditional probability calculations. The values of α and β dictate the number of iterations necessary for classification as can be seen from Fig. 16, Fig. 17. When α and β are closely aligned, fewer iterations are required to generate results. However, a balance must be struck to ensure an adequate number of iterations. Insufficient iterations may lead to inaccurate probability classifications.

The sensitivity of our algorithm becomes apparent when considering the discrepancy between α and β. A greater difference between these parameters results in more precise membership assignments to the positive and negative regions. Thus, careful consideration of the relationship between α and β is crucial in optimizing the algorithm's performance. For situations demanding high accuracy, one should choose a larger difference between α and β to ensure a more precise classification, even if it means more computation time.

On the other hand, if faster processing is crucial, a smaller difference can be used, but that might lead to a potential accuracy reduction.

## 6 Conclusion

In conclusion, the Generalized Sequential Decision-Theoretic Rough Set (GSeq-DTRS) represents a significant advancement in decision-theoretic rough sets (DTRSs). Operating iteratively across multiple levels, GSeq-DTRS effectively addresses the limitations of its predecessor, Seq-DTRS, by managing sequential procedures and flawlessly utilizing complete information. The introduction of generalized granulation, departing from conventional structures to incorporate similarity/tolerance relations, further enhances its capabilities. GSeq-DTRS demonstrates remarkable versatility in handling both continuous-scale and discrete datasets, facilitated by its Generalized Granular Structure (GGS). Additionally, the refinement of conditional probability (CP) aligned with tolerance classes enhances the approach's efficacy. Extensive experimental analysis underscores GSeq-DTRS's effectiveness in accurately classifying elements into positive (POS) or negative (NEG) regions, with lower sensitivity to parametric values and faster convergence compared to traditional Seq-DTRS. Overall, GSeq-DTRS holds promise for enhancing decision-making processes across various domains.

Further research will be focused on developing more efficient algorithms for GSeq-DTRS to handle larger datasets and complex decision-making scenarios. Improving the robustness of GSeq-DTRS against imbalanced and noisy datasets also needs further investigation.

## Data availability

No data available.

## CRediT authorship contribution statement

**Tanzeela Shaheen:** Data curation, Conceptualization. **Hamrah Batool Khan:** Writing – review & editing, Writing – original draft, Methodology. **Wajid Ali:** Investigation, Formal analysis, Data curation, Conceptualization. **Shaheryar Najam:** Validation, Software. **Md. Zia Uddin:** Visualization, Resources, Methodology, Data curation. **Mohammad Mehedi Hassan:** Resources, Funding acquisition, Formal analysis.

## Declaration of competing interest

The authors declare that they have no known competing financial interests or personal relationships that could have appeared to influence the work reported in this paper.
